# 555. Incidence of Postoperative Nausea and Vomiting in Burn Patients Following Monitored Anesthesia Care

**DOI:** 10.1093/jbcr/irag033.295

**Published:** 2026-04-06

**Authors:** Anthony Kovac, Claire E Meeds, Dhaval Bhavsar, Fatma O Sayali, Niaman Nazir

**Affiliations:** University of Kansas Medical Center, Kansas City, KS; University of Kansas Medical Center, Collierville, TN; The University of Kansas Health System, Kansas City, KS; University of Kansas Medical Center, Kansas City, KS; University of Kansas Medical Center, Kansas City, KS

## Abstract

**Introduction:**

The overall incidence of postoperative nausea and vomiting (PONV) observed 2 to 24 hours following general anesthesia may range from 25 to 30% and is commonly due to volatile inhalation agents and opioid analgesics. Besides patient dissatisfaction, PONV can delay recovery and discharge from anesthesia care. PONV is less frequent after monitored anesthesia care (MAC) anesthesia. Burn patients may receive MAC for burn dressing change. There has been minimal research about the incidence of PONV in burn patients following anesthesia. To our knowledge, this may be one of the first reports of PONV incidence following MAC for burn dressing change. Objective: To investigate the incidence of PONV following MAC for patients undergoing burn dressing change.

**Methods:**

Following IRB approval, the electronic medical record (EMR) of burn patients undergoing MAC anesthesia from January 1, 2016, to December 31, 2024, in our burn unit were retrospectively reviewed. Patient demographics, burn injury etiology, comorbidities, TSBA%, opioids, prophylactic antiemetics, and type and dose of medications administered for MAC were analyzed. The primary outcome was the incidence of PONV documented in the EMR within two hours immediately post dressing change prior to discharge from anesthesia.

**Results:**

EMR data of 207 burn patients undergoing MAC were evaluated. PONV occurred in 5 of 207 (2.4%) patients. Comparing patient demographics in the PONV versus no PONV groups, there was no difference regarding age, BMI, and TBSA%. In the PONV group, the mean age (+/- SD) was 40.2 +/- 13.9 years, BMI 27.5 +/- 4.1, and TBSA% 14.9 +/- 11.9. Caucasian males were the largest gender group. The most common physical class was ASA 3: 3/5 (60%) in patients with PONV, and 105/202 (52%) in patients without PONV. Propofol was the most common sedative medication, followed by ketamine and dexmedetomidine. Opioids included fentanyl and hydromorphone. Antiemetics included ondansetron and dexamethasone.

**Conclusions:**

MAC anesthesia can be used for patients undergoing burn dressing change. The incidence of PONV in the two-hour period immediately following MAC anesthesia was low at 2.4%. Male gender and the use of propofol, ketamine and prophylactic antiemetics are possible factors contributing to the low incidence of PONV following MAC anesthesia for dressing change. This is an area of research that deserves further attention.

**Applicability of Research to Practice:**

The incidence of PONV following MAC anesthesia for burn dressing change is low. Medication choice can help influence and decrease the incidence of PONV. Use of sedative medications with low antiemetic potential, such as propofol, prophylactic antiemetics, such as ondansetron and dexamethasone, and use of ketamine to help minimize opioids are important considerations in these patients.

**Funding for the study:**

N/A.

